# Association of IFNAR2 and TYK2 with COVID-19 pathology: current and future

**DOI:** 10.3389/fimmu.2024.1462628

**Published:** 2024-09-16

**Authors:** Alireza Razavi, Maedeh Raei, Ken Shirato

**Affiliations:** ^1^ Student Research Committee, School of Medicine, Mazandaran University of Medical Sciences, Sari, Iran; ^2^ Research Center for Clinical Virology, Tehran University of Medical Science, Tehran, Iran; ^3^ Gastrointestinal Cancer Research Center, Non-Communicable Diseases Institute, Mazandaran University of Medical Sciences, Sari, Iran; ^4^ Department of Molecular Predictive Medicine and Sport Science, Kyorin University Faculty of Medicine, Mitaka, Japan

**Keywords:** coronavirus disease 2019, prognostic factor, tyrosine kinase, interferon alpha-beta receptor subunit 2 (IFNAR2), SARS coronavirus 2

## 1 Introduction

Determining prognostic factors in COVID-19 is important in triaging patients in critical situations (1, 2). In COVID-19, expression of interferon α/β receptor subunit (*IFNAR*) 2 and tyrosine kinase 2 (*TYK2*) has been suggested to be associated with COVID-19 outcomes. To offer potential therapeutic strategies and contribute to better patient care and treatment decisions, we would like to summarize the current findings on the association of *IFNAR2* and *TYK2* genes with COVID-19 pathology and propose future challenges.

## 2 Blockage of IFNAR signaling

Recent genome-wide association studies have shed significant light on the potential involvement of *IFNAR2* and *TYK2* in the dynamics of SARS-CoV-2 infection (3–5). Increased expression of *IFNAR2* has been correlated with a decreased probability of developing critical COVID-19 (3, 6). When type I interferons (IFN-α/β) bind to IFNAR2, it triggers the JAK-STAT signaling pathway, leading to the activation of IFN-stimulated genes (ISGs) that produce antiviral proteins. The activation of ISGs results in the production of proteins that inhibit viral replication and enhance the immune response. This response helps to control the spread of the virus within the host, providing a crucial defense mechanism against COVID-19 (7). Therefore, genetic variations in the *IFNAR2* gene, such as rs2236757 and rs3153, can alter the structure and function of the IFNAR2 protein. These polymorphisms may lead to reduced expression of the receptor or changes in its binding affinity for IFNs. Individuals with certain *IFNAR2* polymorphisms have a higher risk of severe COVID-19 and increased mortality due to impaired antiviral response (8). Furthermore, TYK2 expression is involved in the regulation of IFNAR signaling, indicating its potential significance in disease management (6). Our published work reveals a substantial decrease in the mRNA expression levels of *IFNAR2* and *TYK2* in peripheral blood leukocytes among individuals with COVID-19 (1). Despite the established role of IFNs being induced during viral infections and limiting viral replication through IFN receptor signaling, it is noteworthy that severe cases of COVID-19 display significantly reduced IFN levels compared to other viral infections (9). Lower levels of IFNAR2 can impair the body’s ability to respond to viral infections, leading to more severe disease outcomes. IFNAR2 exists in three isoforms, two of which are soluble but lack the ability to activate signaling upon interaction with type I IFN. Consequently, the efficacy of type I IFN action may be influenced by the relative abundances of these IFNAR2 isoforms, as proposed by Aliaga-Gaspar and colleagues in 2021 (10). In severe COVID-19 cases, higher levels of soluble IFNAR2 (sIFNAR2) have been observed, which can bind to IFNs and prevent them from interacting with cell surface receptors, thus impairing the signaling pathway (11). Coronaviruses, skilled at hiding viral RNA from pattern recognition receptors, may employ these strategies to enable covert replication. The virus interacts with specific proteins to impede IFN responses, avoid detection, or directly block IFNAR signaling. Hence, additional research on *IFNAR2* and *TYK2* expression in COVID-19 can significantly contribute to finding a conclusive treatment for this worldwide crisis.

## 3 Discussion

Our study did not establish that the expressions of *IFNAR2* and *TYK2* genes serve as a predictor of severity of COVID-19, likely due to limitations such as small sample size (1). However, prior evidence suggests that *IFNAR2* and *TYK2* variants could be linked to disease severity due to their high affinity to type I IFNs (3, 12–14). Additionally, measuring soluble IFNAR levels may offer insights into predicting disease severity or mortality risk (12). The most intriguing study conducted by Pairo-Castineira et al. involved a prospective multicenter investigation. They analyzed 2,244 critically ill cases across 208 UK intensive care units, encompassing patients of European, South Asian, African, and East Asian descent. The study focused on the *IFNAR2* gene located on chromosome 21q22.1 and a gene proximal to *TYK2* on chromosome 19p13.2. Their findings indicated that low *IFNAR2* gene expression (OR = 1.28, *P* = 4.99 × 10^-8^) or high *TYK2* gene expression (OR = 1.6, *P* = 2.3 × 10^-8^) correlates with life-threatening conditions (3). A study conducted in a Mexican cohort investigated *IFNAR2* variants (rs2236757, rs1051393, rs3153, rs2834158, and rs2229207) using Taqman^®^ assays in 1,202 patients with severe COVID-19. The findings revealed an association between four of these five variants (rs2236757, rs1051393, rs3153, and rs2834158) and mortality risk among severe COVID-19 patients (15). Consequently, it can be inferred that some *IFNAR2* variants may negatively impact the antiviral effects of IFN α/β (16). Our study revealed that *IFNAR2* and *TYK2* mRNA expressions were significantly downregulated in COVID-19 patients compared to healthy subjects. The discrepancy that our results did not support upregulation of *TYK2* expression may be due to differences in sample size and ethnicity among populations (1). However, at least, impaired IFNAR signaling due to decreased *IFNAR2* expression appears to be associated with COVID-19 severity, as supported by various studies. Specifically, we observed a negative correlation between the expression levels of *IFNAR2* and *TYK2* transcripts in COVID-19 patients (1). This suggests that altered *TYK2* expression may impact immune responses during infection. Further investigation of this issue can be considered as a therapeutic goal.

A recent systematic review summarized that *IFNAR2* variants (specifically rs9976829, rs2834158, and rs3153) are significantly linked to mortality risk (13, 15, 17). Also, variants rs17860118 and rs2229207 in the *IFNAR2* gene have been conclusively linked with susceptibility to SARS-CoV-2 in COVID-19 patients (OR = 1.718, CI 95% = 1.039-2.841, *P* = 0.033, and OR = 1.89, CI 95% = 1.141-3.156, *P* = 0.012, respectively) (13, 18). Based on this recent review, the rs2236757 genetic variant is connected to severe cases of COVID-19 (3, 13, 19, 20). Moreover, the cohort analysis of 694 Brazilian COVID-19 patients reveals a significant link between rs2236757/*IFNAR2* and rs2304256/*TYK2* polymorphisms and worsened COVID-19 outcomes, particularly affecting female and non-white patients (19). Notably, in non-white patients, having both the minor alleles of rs2236757 (*IFNAR2*) and rs12329760 (*TMPRSS2*) leads to an additive increase in the risk of death (19). It appears that this variant interacts with both *TMPRSS2* and *ACE1*. ACE2 acts as the cellular gateway for SARS-CoV-2, while TMPRSS2 facilitates the virus’s entry by activating its spike proteins (21). Genetic variations confidently interact differently with factors such as sex and ethnicity to influence the severity of COVID-19 (1). The virus replicates, causing extensive tissue damage and leading to an overwhelming immune response known as cytokine storm (22). This response occurs as the immune system strives to contain viral replication and handle dying and dead cells. This highlights the urgent need for further research and targeted interventions to address these disparities.

Various studies have reported different findings. In a prospective population-based cohort study conducted in the United Kingdom, researchers collected genetic and phenotypic data from individuals aged 40 to 69 years (23). Specifically, ten phenotypes were associated with a genetic variant called rs74956615 (*TYK2*), all showing reduced odds related to the COVID-19 risk allele. These phenotypes included psoriatic arthropathy (OR, 0.31; 95% CI, 0.20–0.47; q=4.5×10−5), rheumatoid arthritis (OR, 0.83; 95% CI, 0.64–0.83; q=0.0003), and thyrotoxicosis (OR, 0.77; 95% CI, 0.68–0.87; q=0.01). Additionally, seven phenotypes were nominally validated in the CATHGEN study, including psoriasis, rheumatoid arthritis, and hypothyroidism (all *P* < 0.1) (23). These COVID-19-related genetic variants underscore the significance of host antiviral defense mechanisms and inflammatory signaling. TYK2’s involvement in psoriasis is linked to Th17 responses and IFN-α signaling. Notably, the study clarified previously conflicting associations for autoimmune diseases, revealing a novel observation: decreased odds of psoriasis associated with rs74956615, suggesting a distinct impact of this allele on *TYK2* gene function compared to prior genome-wide association study analyses of psoriasis.

An in-depth genetic analysis of 109 patients with PCR-confirmed SARS-CoV-2 infection in Morocco has produced compelling results. Logistic regression models have demonstrated that there are no statistically significant differences in the SNPs *IFNAR2* (rs2236757) and *TYK2* (rs74956615) between patients requiring intensive care and those not hospitalized (*P > 0.05*) (24). The observed variances between this study and other findings could potentially stem from ethnic disparities across countries, highlighting noteworthy population-based distinctions (1). Nonetheless, it is crucial to acknowledge that the disparities in our results compared to those of previously cited studies could also be influenced by interactions with other genetic variations or the effects of distinct risk and protective factors within each population (1).

The COVID-19 pandemic has exerted a widespread global impact, prompting numerous nations to investigate the expression of *IFNAR2* and *TYK2* genes. Notably, the available data primarily encompasses individuals of European ancestry, thereby presenting a limitation in the scope of research. The research findings, including our study as well as other research, underscore the notable impact of small sample sizes. Studies with insufficient sample sizes carry the risk of yielding false-negative results, as researchers may overlook existing effects. The dissemination of false negatives not only distorts theoretical comprehension but also impedes scientific advancement. Furthermore, underpowered studies pose a threat to the reliability of research outcomes, emphasizing the critical importance of meticulously considering sample size during study design. Also, control of confounding factors is crucial for obtaining reliable results.

The Omicron variant (B.1.1.159) of SARS-CoV-2 has raised concerns due to its extensive spike protein mutations, particularly in the receptor-binding domain (RBD) and the N-terminal domain (NTD), which are primary targets of neutralizing antibodies (25). Studies have revealed that SARS-CoV-2 variants of concern, including the Omicron variant, can reduce the expression of major histocompatibility complex class I (MHC-I) molecules (26). These MHC-I molecules are vital for presenting viral antigens to CD8+ T cells, which play a crucial role in immune surveillance and defense against infections (26). SARS-CoV-2 variants can potentially modulate angiotensin-converting enzyme 2 (ACE2) expression levels, which are crucial in viral entry. Any changes in ACE2 expression could significantly impact the severity of infection (27). In fact, ACE2 serves as the primary receptor for SARS-CoV-2, binding of the virus to ACE2 facilitates cell entry. So Increased ACE2 expression can enhance viral entry (28, 29). Consequently, infected cells may face challenges in effectively presenting viral antigens to the immune system due to factors such as the downregulation of MHC-I, which impairs antigen presentation and allows the virus to evade immune surveillance (26).

Some research groups, including us (30), have experimentally demonstrated *in vitro* and *in vivo* that spike proteins have pro-inflammatory properties. Among them, an intriguing study by Li et al. (31) compared the strength of action of wild-type and Omicron spike protein on several immunological parameters. It showed that the ability of the Omicron spike protein to increase the transcriptional activity of nuclear factor-κB and the concomitant production of tumor necrosis factor-α, interleukin-6, and monocyte chemoattractant protein-1 in macrophages was lower than that of wild-type spike protein, which may be one factor contributing to the milder virulence of the Omicron strain. However, we have yet to see studies assessing the impact of SARS-CoV-2 variants on the expression of *IFNAR2* and *TYK2* genes, and this limitation is also present in our research. Further, it is reasonable to assume that unique mutations in non-structural protein (NSP) such as NSP3, NSP6, and NSP13, other main structural protein such as M protein, and accessary protein such as open reading flame 7b (ORF7b) and ORF9b may disrupt the expression of *IFNAR2* and *TYK2* by affecting viral pathogenesis and evading the host’s immune response (32).

Our study was conducted during the Omicron outbreak, but facility limitations prevented us from identifying the specific variant of SARS-CoV-2 responsible for COVID-19 development in patients during that time (1). Given the lack of literature on the impact of the Omicron variant on the expression of *IFNAR2* and *TYK2*, it is crucial to initiate focused projects for further understanding. Additionally, investigating the association between the temporal course of expression changes of these genes and COVID-19 prognosis after infection with wild-type and mutant strains of SARS-CoV-2 would provide valuable insights. Furthermore, analysis using human ACE2-transgenic rodent experimental models in which *IFNAR2* and *TYK2* genes are deleted or overexpressed in a cell-selective manner may also be useful for better understanding the functional mechanisms of these genes in the pathogenesis of COVID-19 caused by wild-type and mutant strains of SARS-CoV-2. It will be necessary to constantly update the above knowledge whenever major mutations that change the properties of the virus occur in the future.

## References

[1] Razavi A Raei M Hatami Y Chokami GS Goudarzi Y Ghasemian R. Evaluation of IFNAR2 and TYK2 transcripts’ prognostic role in COVID-19 patients: a retrospective study. Front Cell Infection Microbiol. (2024) 14:1356542. doi: 10.3389/fcimb.2024.1356542 PMC11089198 38741892

[2] Shojaei M Shamshirian A Monkman J Grice L Tran M Tan CW. IFI27 transcription is an early predictor for COVID-19 outcomes, a multi-cohort observational study. Front Immunol. (2022) 13:1060438. doi: 10.3389/fimmu.2022.1060438 36685600 PMC9850159

[3] Pairo-Castineira E Clohisey S Klaric L Bretherick AD Rawlik K Pasko D. Genetic mechanisms of critical illness in COVID-19. Nature. (2021) 591:92–8. doi: 10.1038/s41586-020-03065-y 33307546

[4] Niemi MEK Karjalainen J Liao RG Neale BM Daly M Ganna A. Mapping the human genetic architecture of COVID-19. Nature. (2021) 600:472–7. doi: 10.1038/s41586-021-03767-x PMC8674144 34237774

[5] Ferreira LC Gomes CEM Rodrigues-Neto JF Jeronimo SMB. Genome-wide association studies of COVID-19: Connecting the dots. Infection Genet Evolution: J Mol Epidemiol Evolutionary Genet Infect Dis. (2022) 106:105379. doi: 10.1016/j.meegid.2022.105379 PMC9584840 36280088

[6] Velavan TP Pallerla SR Rüter J Augustin Y Kremsner PG Krishna S. Host genetic factors determining COVID-19 susceptibility and severity. EBioMedicine. (2021) 72:103629. doi: 10.1016/j.ebiom.2021.103629 34655949 PMC8512556

[7] Raftery N Stevenson NJ. Advances in anti-viral immune defence: revealing the importance of the IFN JAK/STAT pathway. Cell Mol Life Sci: CMLS. (2017) 74:2525–35. doi: 10.1007/s00018-017-2520-2 PMC7079803 28432378

[8] Abdelhafez M Nasereddin A Shamma OA Abed R Sinnokrot R Marof O. Association of IFNAR2 rs2236757 and OAS3 rs10735079 Polymorphisms with Susceptibility to COVID-19 Infection and Severity in Palestine. Interdiscip Perspect Infect Dis. (2023) 2023:9551163. doi: 10.1155/2023/9551163 37745867 PMC10517872

[9] Hadjadj J Yatim N Barnabei L Corneau A Boussier J Smith N. Impaired type I interferon activity and inflammatory responses in severe COVID-19 patients. Science. (2020) 369:718–24. doi: 10.1126/science.abc6027 PMC7402632 32661059

[10] Aliaga-Gaspar P Hurtado-Guerrero I Ciano-Petersen NL Urbaneja P Brichette-Mieg I Reyes V. Soluble receptor isoform of IFN-beta (sIFNAR2) in multiple sclerosis patients and their association with the clinical response to IFN-beta treatment. Front Immunol. (2021) 12:778204. doi: 10.3389/fimmu.2021.778204 34975865 PMC8716373

[11] Yaugel-Novoa M Bourlet T Longet S Botelho-Nevers E Paul S. Association of IFNAR1 and IFNAR2 with COVID-19 severity. Lancet Microbe. (2023) 4:e487. doi: 10.1016/S2666-5247(23)00095-2 37028439 PMC10072862

[12] Montenegro AFL Clementino MAF Yaochite JNU. Type I interferon pathway genetic variants in severe COVID-19. Virus Res. (2024) 342:199339. doi: 10.1016/j.virusres.2024.199339 38354910 PMC10901847

[13] López-Bielma MF Falfán-Valencia R Abarca-Rojano E Pérez-Rubio G. Participation of single-nucleotide variants in IFNAR1 and IFNAR2 in the immune response against SARS-coV-2 infection: A systematic review. Pathog (Basel Switzerland). (2023) 12:1320. doi: 10.3390/pathogens12111320 PMC10675296 38003785

[14] Zabihi Rizi F Ghorbani A Zahtab P Darbaghshahi NN Ataee N Pourhamzeh P. TYK2 single-nucleotide variants associated with the severity of COVID-19 disease. Arch Virol. (2023) 168:119. doi: 10.1007/s00705-023-05729-2 36959416 PMC10035968

[15] Fricke-Galindo I Martínez-Morales A Chávez-Galán L Ocaña-Guzmán R Buendía-Roldán I Pérez-Rubio G. IFNAR2 relevance in the clinical outcome of individuals with severe COVID-19. Front Immunol. (2022) 13:949413. doi: 10.3389/fimmu.2022.949413 35967349 PMC9374460

[16] Fricke-Galindo I Martínez-Morales A Pérez-Rubio G Buendía-Roldán I Chávez-Galán L Hernández-Zenteno R. Relevance of IFNAR2 variants in the mortality of patients with severe COVID-19. Eur Respir J. (2022) 60:2163. doi: 10.1183/13993003.congress-2022.2163

[17] Ma Y Huang Y Zhao S Yao Y Zhang Y Qu J. Integrative genomics analysis reveals a 21q22.11 locus contributing risk to COVID-19. Hum Mol Genet. (2021) 30:1247–58. doi: 10.1093/hmg/ddab125 PMC8136003 33949668

[18] Nhung VP Ton ND Ngoc TTB Thuong MTH Hai NTT Oanh KTP. Host genetic risk factors associated with COVID-19 susceptibility and severity in Vietnamese. Genes. (2022) 13:1884. doi: 10.3390/genes13101884 36292769 PMC9601961

[19] Dieter C de Almeida Brondani L Lemos NE Schaeffer AF Zanotto C Ramos DT. Polymorphisms in ACE1, TMPRSS2, IFIH1, IFNAR2, and TYK2 genes are associated with worse clinical outcomes in COVID-19. Genes. (2022) 14:29. doi: 10.3390/genes14010029 36672770 PMC9858252

[20] Horowitz JE Kosmicki JA Damask A Sharma D Roberts GHL Justice AE. Genome-wide analysis provides genetic evidence that ACE2 influences COVID-19 risk and yields risk scores associated with severe disease. Nat Genet. (2022) 54:382–92. doi: 10.1038/s41588-021-01006-7 PMC9005345 35241825

[21] Sahranavard-Pirbazari P Khoshghiafeh A Kamali MJ Esfandiar H Bakhtiari M Ahmadifard M. A comprehensive review of ACE2, ACE1, TMPRSS2 and IFITM3 gene polymorphisms and their effect on the severity of COVID-19. Adv Med Sci. (2023) 68:450–63. doi: 10.1016/j.advms.2023.10.010 37926001

[22] Brodin P. Immune determinants of COVID-19 disease presentation and severity. Nat Med. (2021) 27:28–33. doi: 10.1038/s41591-020-01202-8 33442016

[23] Regan JA Abdulrahim JW Bihlmeyer NA Haynes C Kwee LC Patel MR. Phenome-wide association study of severe COVID-19 genetic risk variants. J Am Heart Assoc. (2022) 11:e024004. doi: 10.1161/JAHA.121.024004 35179038 PMC9075057

[24] Benmansour R Tagajdid M El Hamzaoui H Fjouji S Doghmi N Houba A. TYK2, IFITM3, IFNAR2 and OAS3 single-nucleotide polymorphisms among severe COVID-19 ICU patients in Morocco. Int J Immunopathol Pharmacol. (2024) 38:3946320241257241. doi: 10.1177/03946320241257241 38760017 PMC11102656

[25] Shao W Zhang W Fang X Yu D Wang X. Challenges of SARS-CoV-2 Omicron Variant and appropriate countermeasures. J Microbiol Immunol Infection. (2022) 55:387–94. doi: 10.1016/j.jmii.2022.03.007 PMC9040366 35501267

[26] Moriyama M Lucas C Monteiro VS Iwasaki A. Enhanced inhibition of MHC-I expression by SARS-CoV-2 Omicron subvariants. Proc Natl Acad Sci United States America. (2023) 120:e2221652120. doi: 10.1073/pnas.2221652120 PMC10120007 37036977

[27] Bourgonje AR Abdulle AE Timens W Hillebrands JL Navis GJ Gordijn SJ. Angiotensin-converting enzyme 2 (ACE2), SARS-CoV-2 and the pathophysiology of coronavirus disease 2019 (COVID-19). J Pathol. (2020) 251:228–48. doi: 10.1002/path.5471 PMC7276767 32418199

[28] Pouremamali A Babaei A Malekshahi SS Abbasi A Rafiee N. Understanding the pivotal roles of ACE2 in SARS-CoV-2 infection: from structure/function to therapeutic implication. Egyptian J Med Hum Genet. (2022) 23:103. doi: 10.1186/s43042-022-00314-9 PMC9206724 37521846

[29] Saponaro F Rutigliano G Sestito S Bandini L Storti B Bizzarri R. ACE2 in the era of SARS-coV-2: controversies and novel perspectives. Front Mol Biosci. (2020) 7:588618. doi: 10.3389/fmolb.2020.588618 33195436 PMC7556165

[30] Shirato K Kizaki T. SARS-CoV-2 spike protein S1 subunit induces pro-inflammatory responses via toll-like receptor 4 signaling in murine and human macrophages. Heliyon. (2021) 7:e06187. doi: 10.1016/j.heliyon.2021.e06187 33644468 PMC7887388

[31] Li X Li W Liu Z Kang Y Zhang X Xu Z. A comparative study of spike protein of SARS-CoV-2 and its variant Omicron (B. 1.1. 529) on some immune characteristics. Sci Rep. (2022) 12:17058. doi: 10.1038/s41598-022-21690-7 36224298 PMC9554390

[32] Hossain A Akter S Rashid AA Khair S Alam ARU. Unique mutations in SARS-CoV-2 Omicron subvariants’ non-spike proteins: Potential impacts on viral pathogenesis and host immune evasion. Microbial Pathogenesis. (2022) 170:105699. doi: 10.1016/j.micpath.2022.105699 35944840 PMC9356572

